# The next generation of malaria treatments: the great expectations

**DOI:** 10.2144/fsoa-2023-0018

**Published:** 2023-03-09

**Authors:** Berjas Abumsimir, Talal S Al-Qaisi

**Affiliations:** 1 Department of Medical Laboratory Sciences, Pharmacological & Diagnostic Research Centre (PDRC), Faculty of Allied Medical Sciences, Al-Ahliyya Amman University (AAU), Amman, 19328, Jordan

**Keywords:** chloroquine, hemozoin, malaria, plasmodium, vaccine

This commentary aims to provide a concise scientific review of the major treatments of malaria and to assess the biological mechanisms of action of these treatments at the molecular level, old ones, as well as those in the development and trial phases. We also discuss the current trends in developing treatments and vaccines based on a clear understanding of the biological observations associated with *Plasmodium spp.* and its response mechanisms to these drugs.

Malaria is one of the big three threats (malaria, tuberculosis and HIV) to human health in the modern age. Despite the enormous efforts made to combat the disease, the WHO has released its most recent data, which indicated that there were 619,000 malaria deaths globally in 2021. Currently, active initiations worldwide are in progress in the hope to eliminate malaria. Remarkably, the incidence of malaria infections is increasing, not only in locations where it is endemic but also in areas where notable malaria infections have not been previously noted. The number of malaria cases has increased from 232 million cases in 2019 to 247 million cases in 2021 [1]. This increase could be attributed to many reasons, first; the displacement of people as a result of wars, as well as higher migration movements from Africa to Europe and from Latin and Central America to North America as a result of growing rates of hunger and poverty. Second, climate change could be a key player. Some areas that were previously unexposed to malaria become at risk of malaria transmission since they become more suitable for the growth of *Anopheles* insects that have a predominant role in the transmission of *Plasmodium* parasite. Finally, insecticide resistant *Anopheles* are rapidly evolving due to the uncontrolled use of pesticides by farmers in agriculture.

Untreated malaria infections may cause death, but even with treatment, serious complications of the infections could occur especially in endemic regions such as sub-Saharan countries. The complications of malaria include cerebral malaria leading to coma and death, respiratory distress, enlargement of the spleen and liver and kidney failure. In chronic cases, pregnant women could transmit infections to the embryos resulting in miscarriage, infant mortality and low birth weight [2].

Humans learned by experience how to deal with this disease and to find compounds that could combat it. In general, most malaria treatments are now known to work by inhibiting the growth of the malaria parasite. Among these drugs, is artemisinin, which has been used in China for more than 2000 years. Which prompted contemporary biochemical studies to investigate its mechanism of action against malaria symptoms. artemisinin is assumed to inhibit the growth of the compound hemozoin, which is generated by the malaria parasite. The malaria parasite, like all blood-feeding parasites, leaves free heme as a toxic byproduct of the feeding process. Parasites developed a mechanism to detoxify the free heme by nucleation in form of crystals called hemozoin [3].

Drugs, such as artemisinin and mefloquine, block this process and inhibit parasite growth [4]. But it is not that simple, as there are many questions about the explanations for the mechanism of action of these drugs. Such as the inconsistency between the degree of solubility of these compounds in water and the extent of their effect on the process of crystallizing hemozoin, which takes place within lipid droplets inside the digestive vacuole of the parasite. Some argue that fragments or parts of hemozoin may interact with these drugs, partially inhibiting parasite reproduction and; therefore, relieving the symptoms of malaria [5].

We have to point out here that one of the most important strategies to combat infection with the malaria parasite is to stop hemozoin crystallization. There are many studies involved in describing the mechanism of action of these drugs. New generations of these drugs are being developed, which we will discuss later in this article.

These drugs have earned great popularity for various reasons, the most significant of which are their high antimalarial efficacy, low toxicity and low cost. However, despite this, there are harmful side effects for some of these drugs, particularly chloroquine. More crucially, in my opinion, the ambiguity still surrounds the mechanism of action of this unique drug. Especially, since it is used as well to treat other human diseases.

Chloroquine is an old treatment not only for malaria but also for some autoimmune diseases, and it has been used partially to treat cancer [6]. Additionally, it was also used to treat SARS-CoV-2 infections in the 2020 pandemic [7]. For all these life-threatening diseases, the mechanism of action of chloroquine is still controversial. In malaria, the suggested mechanism of action is interference with hemozoin crystallization. Those who support this mechanism used physiochemical approaches to prove the high affinity of chloroquine to hemozoin which is supported by the relative efficiency of chloroquine against *Plasmodium spp.* However, this suggested mechanism does not answer the question: how does a poorly lipid-soluble chloroquine reach and inhibit hemozoin formation inside the digestive vacuole of the parasite which is filled with lipids and covered by the lipid layers?

In addition, if the interference of hemozoin crystallization is the correct mechanism; what about the progressively resistant strains of *Plasmodium* (the first reports of such resistance were since 1950)?

The answer was that resistant strains can remove the chloroquine drug from the digestive vacuole using a transmembrane pump. Resistant strains are designated as *P. falciparum* chloroquine resistance transporter (*PfCRT*) gene, ABC transporter *P. falciparum* multidrug resistance (*PfMDR1*) gene and altered chloroquine-transporter protein, *CG2* has been associated with chloroquine resistance. Subsequently, a treatment combination of chloroquine with other drug agents has been proposed to avoid this resistance [8,9].

This proposed mechanism is not the only one, among other suggested mechanisms: inactivation of heme-polymerase, weakening hydrogen bonds in DNA, impairment of metabolic processes, damage of the parasite’s biological membranes, inhibition of DNA and RNA polymerase of the parasite or an anti-inflammatory role [10,11].

Increased resistance to chloroquine among other factors is a great motivation to search for a more ideal treatment. There are already some similar compounds such as artemisinin, mefloquine or ferroquine, that work hypothetically as chloroquine against heme biocrystallization. But the challenge here is to change the approach (targeting hemozoin) instead of searching for similar chloroquine derivatives.

Sooner or later, the classical treatments for malaria will be less efficient or ineffective. The level of prevention such as vaccination and curation is on the way to producing the next generation of malaria treatment. All treatments or vaccines of course are based on a deep knowledge of the parasite life cycle, interaction with the immune system, hemozoin nucleation mechanisms and *Plasmodium spp.* interspecies capabilities resistant against chloroquine and similarly acting compounds.

Let us now briefly review the most promising vaccines and drugs that are being used or those in the process of being used against malaria. At present, many vaccines are based on live, attenuated *P. falciparum* parasites (sporozoites), obtained from the salivary glands of mosquitoes. The attenuated (chemically, genetically or by radiation) *P. falciparum* sporozoite (PfSPZ) is the recent type of these vaccines. The PfSPZ vaccine consists of active, attenuated sporocysts that migrate to liver cells where CD8^+^ T cells that produce IFNγ are activated. The frequency of PfSPZ-specific CD3^+^CD4^+^, CD3^+^CD8^+^ and CD3^+^γδ T cells is dose-dependent [12]. The successful vaccine should be efficient and durable and pass a series of clinical trials. Developed by the company Sanaria, the results were safe and extremely tolerated and efficient. At the 6 month follow-up, vaccine efficacy was up to 48%. At the 18 month follow-up, vaccine efficacy was up to 46% and complete protection was observed after 10 weeks with three doses of PfSPZ-CVac [13].

Another option in malaria vaccination is the use of recombinant protein as a stimulant to the immune system, mostly this type of recombinant protein is used with adjuvant stimulants [14]. The problem with this parasite is its wonderful ability to evade the immune system through a variety of mechanisms. The human immune system produces many types of antibodies against malaria, but these defense tools are not sufficient despite many repeated infections. Thus, choosing the perfect antigen is critical to make immune system memory to prevent parasite evasion mechanisms. On this base, the recombinant proteins as vaccines are used currently for these purposes.

One of the currently proposed antimalarials is ganaplacide It belongs to the class of imidazolopiperazines, Unexpectedly, the exact mechanism is unidentified but suspected to be hemozoin inhibition. In addition to some adverse side effects, certain strains of *Plasmodium spp.* are resistant to ganaplacide. Due to the partial efficacy of ganaplacide, it is recommended to combine it with lumefantrine, which has been a classic antimalarial drug in China since the 1970s. The commonality of the two compounds is that both are thought to be hemozoin inhibitors.

Cipargamin is another synthetic molecule used as antimalarial, which structurally imitates GNF439, the latter was identified as a *P. falciparum* ATP4 (PfATP4 protein) inhibitor. With rapid efficacy in killing the parasite, cipargamin could potentially be used as a treatment for malaria soon [15].

Ivermectin is one of the controversial compounds. It has been known for decades as an antiparasitic agent but not specifically for malaria. Now, this compound and its derivatives are being investigated as a prospective antimalarial based on its action by binding the glutamate-gated chloride channel of the parasite tissues causing the channels to open and disrupt the parasite cell membrane’s polar stability [16]. But until now, no notable efficacy trials have been done against malaria.

Atovaquone is a hydroxy-1,4-naphthoquinone, which is recognized as an antimicrobial, an anti-parasitic (certainly for *Toxoplasma gondii*), and is also used as an antimalarial. The mechanism of action is not completely described. But it is thought to inhibit the mitochondrial electron transport chain of the parasite. Usually, atovaquone is used in combination with proguanil (chlorguanide) which acts as an inhibitor of dihydrofolate reductase [17]. GABA-A receptor antagonists are also now under investigation to be an antimalarial drug candidate [18].

An exciting notice of most malaria treatments is that the precise mechanism of action is not perfectly understood. This lack of understanding presents a challenge in enhancing the efficacy of antimalarial drug candidates. This shortage of knowledge originates from our poor understanding of some biological behavior of *Plasmodium* itself, certainly the hemozoin biocrystallization. An additional shortage too is our poor understanding of *Plasmodium* neutrophiles–monocytes interactions. Despite this, drugs such as artemisinin and chloroquine are very old medications, and of course malaria, itself is a well-known disease.

Separately from vaccination, the description of new compounds against malaria depends on experimentation of the disease manifestation not understanding the behavior of *Plasmodium* and the phenomena related to it. Two phenomena should be elucidated very well before the dream of malaria elimination: the hemozoin biocrystallization and *Plasmodium* interaction with neutrophils and monocytes. And since we can target the hemozoin nucleation with unique compounds in the infected patients, this will be the best choice for treatment.

Targeting any other parts of the *Plasmodium* such as ion channels of the parasite or the nerve receptors of parasites will not be specific and consequently result in adverse side effects for the infected persons. The specificity of the candidate compound treating malaria should be based on the biological specific characteristics of this parasite.

The phenomenon of feeding on hemoglobin is not exclusive to *Plasmodium*, as it is a common feature of many protozoa and arthropods. Advancement has been achieved exploring the hemozoin nucleation mechanisms, but this is not sufficient. It is necessary to study this phenomenon carefully in comparative studies within *Plasmodium* species and within hemoglobin–feeding parasites, to know the mechanisms of drug–hemozoin binding as a method of inhibiting the growth of the parasite.

What’s more, it is important to investigate the exact structural transformations of this parasite in its different life stages in mosquitoes and humans. In future, it will be vital to probe the biological mechanisms of the parasitic apicoplast, as the organelle demonstrates the ability to circumvent hemozoin toxicity? Full comprehension of this process on a molecular level will open up new druggable targets and advance antimalarial drug discovery.
